# Outbreak of Hepatitis A in a Nursery School

**DOI:** 10.1155/2013/684908

**Published:** 2013-09-19

**Authors:** Antonia Galmes-Truyols, Jaume Gimenez-Duran, Antonio Nicolau-Riutort, Catalina Bosch-Isabel, Juana Maria Vanrell-Berga, Margarita Portell-Arbona

**Affiliations:** Directorate of Public Health, Balearic Islands, Spain

## Abstract

In a background of very low incidence of hepatitis A HA in the last decade (annual average of 1.8 cases per 100,000 inhabitants) we describe an outbreak of HA which evolved in Mallorca between May and August 2010, whose main focus was a nursery school where more cases were parents and other young relatives of the children of the institution. Thirty-four cases were defined as outbreak cases. Ten were children of the nursery or their siblings and 22 adults (3 staff members of the nursery and 19 relatives; median age 33 years). The first detected cases were children of the same class. There were 2 adults with haematological complications, though not severe. All children, nursery staff members, parents, and siblings of the cases of the first affected class were immediately offered HA vaccination, but only 43.3% eligible individuals accepted it. None of the cases had been vaccinated. The outbreak spread mostly from asymptomatic children to young adults, showing the changes in HA pattern. That is of great concern as the risk of severe illness rises with age. This incident shows the need to implement new HA vaccination policies in outbreak control. This was later carried out.

## 1. Introduction

Hepatitis A (HA) is still an important public health issue and highlights the need for increased awareness of both the risk of infection to the individual and the possibility of community outbreaks within a changing epidemiology [1]. The disease is caused by the hepatitis A virus (HAV), which is transmitted primarily via the faecal-oral route, either through ingestion of contaminated food and water or through direct contact with an infectious person.

The incidence of HA is strongly correlated with socioeconomic indicators. Thus, with increasing incomes and access to clean water and adequate sanitation, the incidence of HAV infection decreases [2]. Serological prevalence profiles vary geographically. In most low-income countries the prevalence of anti-HAV antibodies in the population may exceed 90% by the age of 10 years. In those areas, exposure to HAV usually occurs before the age of 5 years when most infections are asymptomatic. As a result, there are few susceptible adolescents and adults and little symptomatic disease.

In high-income countries, the seroprevalence of antibodies is very low, and less than 50% are immune by the age of 30. The high proportion of susceptible individuals among adults could theoretically allow transmission, but circulation of the virus is scarce. However, foodborne outbreaks [3] have occurred, for example, after ingestion of shellfish living in sewage-polluted waters [4] or through contaminated vegetable salads [5]. In addition, HAV infection may occur in individuals or groups with high risk of infection, such as travellers to areas of high endemicity [6], men who have sex with men [7], intravenous drug users, and in other specific subpopulations [8].

A substantial proportion of adolescents and adults are susceptible in most developed countries. These ages are associated with a higher rate of severe clinical manifestations, [9] and in some countries in transition the HAV infection has become the leading cause of fulminant hepatic failure.

In the Balearic Islands the drinking water safety and treatment of sewage water have much improved since the eighties and, as a result, the incidence of HA dramatically decreased. Important changes in epidemiological pattern have been detected. Thus, the annual average in the eighties was around 1,100 cases, 400 in the nineties and less than 20 in the first decade of the 21st century, and no food or waterborne outbreaks have been detected in the last 15 years (data from Surveillance Network of Infectious Diseases in the Balearic Islands). The predominant transmission way has changed from water or food to direct contact, and, consequently, the incidence has decreased and the susceptibility in young and middle-age adults has increased. This was proven by seroprevalence studies conducted in other Spanish regions as Catalonia, a region with great similitude to the Balearic Islands, in 2001 [10] (see Table 1) or the Basque Country in 2011 [11]. This has important implication in terms of complications and severe illness and a higher risk of transmission in specific groups, such as MHSM [12]. Infected children who in most cases have subclinical or very mild illness act as a faecal-oral vehicle to their caregivers, most of whom belonging to susceptible groups. Nowadays, because of these changes, the main affected group is that of young men, and the hospitalisation rates have increased. These changes have led to review of HA control policies, including the use of the HA vaccine to control outbreaks and to prevent major complications in high risk groups [13]. The vaccine has been also included in the children's immunisation schedule in a few countries. In Spain only Ceuta and Melilla, areas of high incidence in the Moroccan border, and Catalonia have included it in their immunisation schedule. In the Balearic Islands, there was no indication to vaccinate contacts to control outbreaks, a measure that has proven useful, but whose usefulness is clearly related to the quickness of the intervention and width of coverage [14, 15].

## 2. Objective

The aim of this paper is to describe an outbreak of HA, which evolved in Mallorca between May and August 2010, whose focus was a nursery school and involved parents and other young relatives of the children.

## 3. Detection of the Outbreak

On 7th of June 2010, a nursery school staff reported four possible cases of HA. Two of them were children attending the same classroom (Classroom A) in the nursery; one was a caregiver and the 4th was father of an asymptomatic child of the same classroom. An epidemiological investigation was initiated, and the first control measures were taken to minimize the virus transmission among the children and their contacts.

## 4. Methods

The case was defined as individual with compatible symptoms, epidemiologically linked to the school (i.e., children and staff members and their domestic or other close contacts) after the 1st of May 2010.

The surveillance of HA was enhanced. The area health services were informed of the outbreak so they could detect possible new cases quickly and were required to report them urgently. The laboratory was also asked to provide the investigation team with a list of positive IgM specific tests for HAV since April to identify nonreported cases and help to reconstruct the outbreak. The nursery staff were asked to report any possible new case.

Epidemiological, clinical, and laboratory data were collected through interviews with the nursery staff and patients (or, if children, to their parents), hospital and primary care centre electronic health records (EHR), and laboratory records. A standardised questionnaire was applied by public health professionals to all the cases detected through medical reports or other cases' interview. Demographic (age, sex, place of residence), clinical (symptoms, onset date, hospitalisation status), and epidemiological characteristics (food questionnaire, contacts with persons diagnosed with HA or with jaundice in the previous 6 weeks, possible link with the nursery, classroom attended by the case or, if the affected person was external to the nursery, the classroom attended by his or her contact). The culture and subsequent viral genotyping were not available, thus no samples were collected for this purpose.

In order to control the outbreak, the nursery staff and the children's families were informed of the outbreak, advised to take hygienic measures to avoid person-to-person transmission and maintain the isolation of cases during the infectious period. Vaccination was offered to all the children and staff of the nursery and domestic contacts of the affected classroom born after 1976 and older than 12 months, provided they had not previously been vaccinated nor had had HA before.

## 5. Results

In the nursery school, there were 61 children aged from 6 months to 3 years and 6 staff members. The children were distributed into classes according to their year of birth (2009, 2008, or 2007), but the staff members took care of children from any classroom. The school received municipal tap water, and the children drank bottled water. Few children ate at the nursery. This fact, added to the presentation of the cases, suggested early person-to-person transmission, so the food questionnaire was only accomplished for the first cases. 

**Figure 1:**
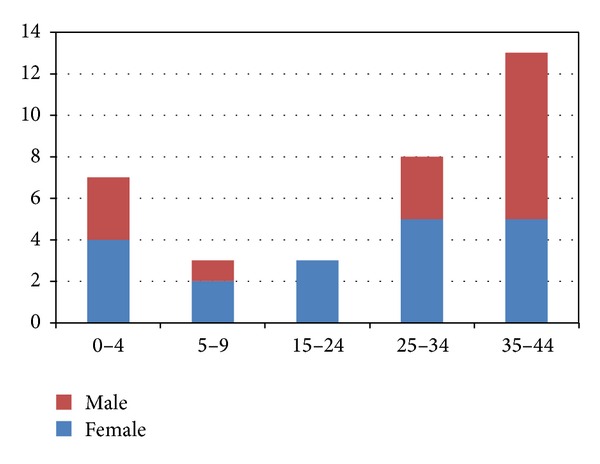
Number of cases by age and gender.

**Figure 2:**
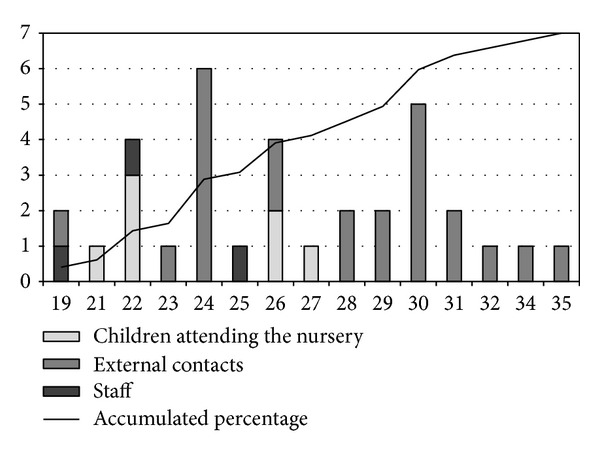
Epidemic curve by week. Number of cases distributed by the epidemiological link to the nursery. Accumulated percentage.

The total number of cases was 34, and most of them were women (19 cases, 55.4%). The mean age was 24 years (SD 15) and the median 29, and the range went from 2 to 41 years.  Figure 1 shows the distribution by gender and age group. Thirty-two cases were confirmed by IgM testing and two others by clinical syndrome and epidemiological link to the nursery. The outbreak lasted for 17 weeks between the 13th of May and the 1st of September, that is, 4-5 generations of cases. See the epidemic curve in Figure 2.

The main part of the cases (23 cases, 70.6%) was external contacts of the children, and most of them were contacts of asymptomatic children (see Table 2). The first cases of the outbreak were linked with classroom A. The index case was the father of an asymptomatic child attending this class, to which fifteen of the cases (5 children, 7 domestic contacts, and the three staff members) were linked. The attack rate was only calculated for the exposed persons in the nursery, as it was impossible to know the exposed persons elsewhere. Overall, it was 14.9% higher among the staff (60%) and the children of classroom A (29.4%) and lower (4.4%) among the rest of them.

The index case was carefully interviewed about food or beverage or contact to a known HA case, but the source of infection could not be detected.

The symptoms were milder in children. Thus, jaundice was present in 30% of children aged up to 14 years old, while it went up to 88% in the cases older than that. The two cases with complications were adults. Both suffered from haemorrhagic complications and they both recovered.

Two of the cases had been vaccinated during the outbreak, both within the 3 weeks prior to the onset of symptoms, so these issues were not considered vaccine failures.

Most of the cases were detected through the specific procedures instituted to reinforce the active surveillance of the HA throughout the outbreak: reporting from laboratory (32.4%) and nursery (20.6%), interviews with the detected cases (17.6%), and some other ways (5.9%). Only 23.5% were notified by the doctors who diagnosed the cases and who were meant to report them.

The vaccination of close contacts of the cases was decided immediately when there were affected children only in the classroom A and implemented on 12th and 13th of June. The eligible persons for vaccination were defined as any person attending or working in the nursery and the domestic contacts of the children from classroom A younger than 45 years old, provided they neither had previously been vaccinated nor had had HA. The total number of eligible persons in the nursery was 62 (4 workers and 58 children, 14 of them attending classroom A). The list of domestic contacts was not obtained, but they were estimated at around 35. Forty-two people received the vaccine (43.3% of all those eligible): 27 in the nursery (1 worker of 4 7 children in classroom A, and 19 from other classrooms) and 15 domestic contacts.

## 6. Conclusions

The absence of any common water or food consumption and the time of the onset of the symptoms of the cases suggest that the transmission way was faecal-oral alone. The index case was suspected of being the primary case for the outbreak, but this is unlikely, as his contact with the children from the nursery was scarce. It seems likely that there could have been prior cases in the nursery, probably mild or asymptomatic, and that one or more of them, probably the son of the index case among them, were responsible for the spread of the HAV. This hypothesis is reinforced by the fact that there is only a 2-day difference between the onset of the two first cases, the index case and a nursery worker, and no other common source could be found for these two cases. The control measures in such a situation have limited effects because when the outbreak is detected the HAV is already circulating among the population and many people can be infected. Besides, the hygienic measures to avoid faecal-oral transmission are not easy to maintain among a population of small children, and the vaccination needs some weeks to develop protection. Regarding the efficacy of the isolation of cases, when the illness is suspected the infectious period has already began. For all these reasons a new generation of cases must be expected after the first public health interventions.

In this outbreak the public health response was quick. Within the first 24 hours after the notification the following measures were taken: the nursery received the instructions to avoid faecal-oral transmission, the families were informed of the outbreak and the hygienic measures, and the health system was informed and given instructions to collaborate in the early detection, diagnosis, and communication of the cases. Within the first week the vaccine administration was implemented. Nevertheless, we must point out some weaknesses of the intervention that most likely diminished its efficacy. First, the outbreak was detected 7 weeks after the onset of the index case because the first cases had not been reported by their doctors. By this time the HAV had already spread among the nursery staff and children, and the Department of Public Health only knew about this when there were already three cases in the nursery after the staff informed about it. Second, the vaccine coverage in children was low, and the highest was that of classroom A, probably a group with a high virus circulation at that time which implies a high percentage of children already infected. Third, the target population included only the domestic contacts of the children of classroom A because when the outbreak was detected all the known cases were linked to this class. The public health team assessed the possibility of widening the target population, but the low response and the logistic difficulties discouraged them.

Current data suggest that there is an important group of susceptible young adults, and it is likely that epidemics will appear in populations at a higher risk of severe complications if infected with HAV. The outcome of this outbreak seems to strengthen this hypothesis.

## 7. Recommendations

The vaccination policy of HA in the Balearic Islands, as well as in the majority of Spain, is limited to some high-risk individuals, as travellers to highly endemic zones or people suffering from several specific pathologies. The vaccine is also used to control outbreaks but not widely and not always easily available. Regarding the use of the vaccine, from our point of view this outbreak is a good example of the convenience of widening the target group when there are children involved. As it was seen later, the virus was already circulating among the children, who acted as asymptomatic diffusers of the HAV towards their families. As the risk of complications is much higher in adults, protection should be seriously considered. The HA vaccine in the Balearic Islands is freely provided by the health system only to persons of risk of complications and that has to be verified by a medical report. Thus, the supply to wide population groups was not easy nor quick, and this was a logistic problem that usually restricted and delayed the use of the vaccine to control outbreaks. That really had to be solved.

After this outbreak, the action protocols for the control of HA outbreaks have changed in the Balearic Islands, including the use of the HA vaccine in close contacts of the cases and widening the circle of eligible persons when the outbreak takes place in settings with small children. An immediate vaccine supply has been warranted to obtain a quick public health response. 

It is also advisable to reinforce the immediate reporting of HA cases to allow a quick investigation of sources of infection and contacts for a further appropriate and timely intervention.

## Figures and Tables

**Table 1:** Percentage of immunity of the population older than 15 years old in Catalonia by age groups, 2001 attending to data of seroprevalence study.

Age group	Year of birth	Positivity antibodies anti-HAV (%)	95% CI
15–24	1977–1986	15.4	9.3–21.5
25–34	1967–1976	35.0	28.7–41.3
35–44	1957–1966	75.1	69.9–80.3
45–54	1947–1956	93.8	90.9–96.6
55–64	1937–1946	97.3	95.2–99.4
≥65	Before 1937	98.2	96.2–100.0

Source: [10].

**Table 2:** Distribution of the cases according to their link to the nursery, by classroom. The cases outside the nursery are distributed according to the presence of symptoms of the contact of the nursery.

Classroom	Children attending the nursery	Staff	Contact of children of the nursery		Total
			Asymptomatic	Symptomatic	
A	5	0	6	1	12
Other	2	0	16	1	19
Any	0	3	0	0	3

Total	7	3	22	2	34

Percentage	20.6	8.8	64.7	5.9	
